# The flavonoid profile of pigeonpea, *Cajanus cajan*: a review

**DOI:** 10.1186/s40064-015-0906-x

**Published:** 2015-03-13

**Authors:** Aaron Nix, Cate A Paull, Michelle Colgrave

**Affiliations:** CSIRO Agriculture Flagship, GPO Box 2583, Brisbane, QLD 4001 Australia

**Keywords:** Flavones, Isoflavones, Flavonols, Flavanones, *Helicoverpa*.spp

## Abstract

**Background:**

Pigeonpea is ranked as the sixth largest grain legume produced by volume and as such is a major global food crop for livestock and human consumption. We show that pigeonpea contains a number of flavonoids and report their distribution and concentration within different parts of the plant.

**Findings:**

There are a total of 27 flavonoids reported in the literature representing seven flavonoid classes. We found no published evidence of flavanols (catechins/flavan-3-ols) or aurones reported from pigeonpea, nor any study of the flavonoids from pigeonpea flowers.

**Conclusions:**

Despite over 40 years of research in to various aspects of pigeonpea we identified research gaps related to the phytochemical properties of pigeonpea. We explain how addressing these gaps could help to realise the full potential of pigeonpea in agricultural production.

## Introduction

Flavonoids are a large group of polyphenolic compounds produced by plants and play important roles within various organs to maintain plant health, development and growth (Falcone Ferreyra et al. 2012). Flavonoids can function as phytoalexins, photoprotectors, and in Leguminosae, *nod* inducers for nitrogen-fixing bacteria. Flavonoids have been shown to affect the feeding behaviour of invertebrate pest species (Green et al. 2003) and flavonoid profiles have also been used in plant chemotaxonomy to elucidate phylogenetic relationships (Emerenciano et al. 2001). Profiling flavonoids has also enabled the validation of the floral origin of honey (Tomas-Barberan et al. 2001) and the ability to identify varieties of sorghum (Dykes et al. 2009).

Pigeonpea, *Cajanus cajan*, [(L.) Millspaugh] is a perennial legume (subfamily Papilionoideae) grown in many developing countries in the semi-arid tropics and subtropics (Zu et al. 2006). *Cajanas cajan* is the sole crop from the subtribe Cajaninae (tribe Phaseoleae). The genus *Cajanus* is comprised of 34 species, of which 17 are from Australia (15 endemic) with most of the remaining species found on the Indian subcontinent (Van der Maesen 1985, 2003). Often cultivated as an annual (Fu et al. 2006), pigeonpea is a major grain legume crop ranked sixth in area and production globally (Fu et al. 2008). It is an important source of protein in human diets used in dhal and as a green vegetable (Saxena 2010; Singh et al. 1984). Dried seeds of pigeonpea are also crushed and used for animal feed (Fu et al. 2008), and more recently transgenic varieties of pigeonpea have enabled the delivery of protective antigens through fodder for livestock (Satyavathi et al. 2003). Pigeonpea is also used as a mandated refuge crop in Australian cotton production to help reduce the likelihood of *Helicoverpa* species developing resistance to *Bt* cotton (Baker and Tann 2013).

Mounting evidence on the biological activities of flavonoids has increased interest in the possible applications of these compounds in medicine and plant/agricultural sciences. Concurrently, with advances in extraction methods (e.g. microwave-assisted, enzyme-assisted, ultrasonic) and detection using high performance liquid chromatography (HPLC) (Chen et al. 2011), there has been an increase in the ability of researchers to identify flavonoids present in plant material. In this paper we distil all known information on the flavonoid profile of pigeonpea, to facilitate further explorations on the chemical ecology of this species and possible interactions with *Helicoverpa armigera.*

### Flavonoids of pigeonpea: within-plant distribution and function

**Figure 1:**
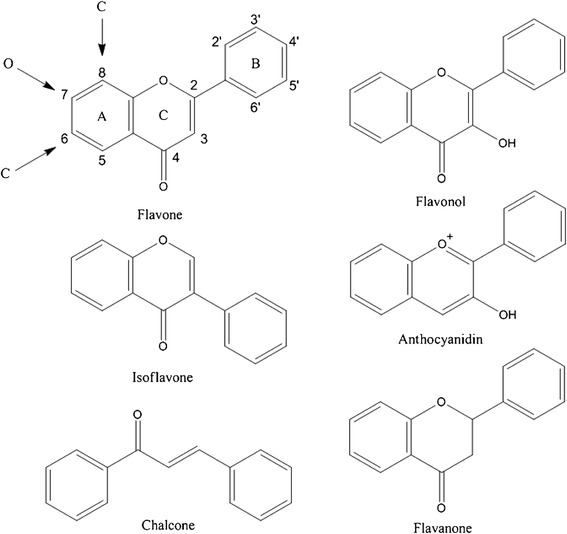
**Generalised flavonoid structure with numbering based on flavone skeleton.**

Flavonoids are found throughout various plant organs in pigeonpea. The biological activities of the flavonoid classes (Figure 1) and their roles in plant defence may be indicative of their distribution throughout the plant (Falcone Ferreyra et al. 2012). A total of 27 flavonoids have been identified, consisting of six flavones, eight isoflavones, four flavonols, two anthocyanins, three flavanones, three isoflavanones and a single chalcone (Table 1).

**Table 1:** **Distribution of flavonoids in** ***Cajanus cajan***

**Flavonoid class**	**Name**	**Structure**	**Organ isolated from**	**Reference**
**Flavones**	Apigenin	5,7,4′-trihydroxyflavone	Leaves	Fu et al. (2008); Zu et al. (2006); Wei et al. (2013a); Wei et al. (2013b)
	Vitexin	Apigenin 8-C-glucoside	Leaves	Fu et al. (2007); Wu et al. (2009)
	Isovitexin	Apigenin 6-C-glucoside	Leaves	Fu et al. (2007)
	Apigenin-6,8-di-C-α-ι-arabinopyranoside		Leaves	Wei et al. (2013b)
	Luteolin	5,7, 3′,4′-tetrahydroxyflavone	Leaves	Fu et al. (2008); Fu et al. (2006); Wei et al. (2013b); Zu et al. (2006)
	Orientin	Luteolin 8-C-glucoside	Leaves	Wei et al. (2013b); Wu et al. (2009)
**Isoflavones**	Biochanin A	5,7-Dihydroxy-4′-methoxyisoflavone	Leaves & roots	Duker-Eshun et al. (2004); Wei et al. (2013a)
	Cajanin	5, 2′,4′-Trihydroxy-7-methoxyisoflavone	Seed & etiolated stems	Dahiya et al. (1984); Ingham (1976) Dahiya (1987)
	4′-O-methylcajanin	5,2′-dihydroxy-7,4′dimethoxyisoflavone	Etiolated stems	Ingham (1976)
	Cajaisoflavone		Root bark	Bhanumati et al. (1979a)
	Formononentin	7-hydroxy-4′methoxyisoflavone	Etiolated stems & leaves	Ingham (1976); Wei et al. (2013a)
	Genistein	5,7,4′-trihydroxyisoflavone	Roots/root bark & etiolated stems	Bhanumati et al. (1979b); Duker-Eshun et al. (2004); Ingham (1976)
	2′-Hydroxygenistein	5,7,2′,4′-tetrahydroxyisoflavone	Roots, etiolated stems	Duker-Eshun et al. (2004) Ingham (1976)
	Isogenistein 7-glucoside	5,7,2′-trihydroxyisoflavone 7-0-β-D-glucoside	Root bark	Bhanumati et al. (1979b)
**Flavonols**	Quercetin	3,5,7,3′,4′-pentahydroxyflavone	Leaves & pod surface	Green et al. (2003); Zu et al. (2006)
	Isoquercitrin	Quercetin 3-β-D-glucoside	Pod surface	Green et al. (2003)
	3-*O*-Methylquercetin	5,7,3′,4′ -Tetrahydroxy-3-methoxyflavone	Pod surface	Green et al. (2003)
	Isorhamnetin	3′-Methoxyquercetin	Leaves	Zu et al. (2006)
**Anthocyanidins**	Chrysanthemin	Cyanidin 3-glucoside	Location not reported	Lai et al. (2012)
	Peonidin 3-glucoside		Location not reported	Lai et al. (2012)
**Flavanones**	Cajaflavanone		Root & root bark	Dahiya (1991); Bhanumati et al. (1978)
	Naringenin	5,7,4′-trihydroxyflavanone	Leaves	Wei et al. (2013b)
	Pinostrobin	5-hydroxy-7-methoxyflavanone	Leaves	Wei et al. (2013a); Wei et al. (2013b); Wu et al. (2009); Ashidi et al. (2010); Duker-Eshun et al. (2004); Kong et al. (2010); Nicholson et al. (2010)
**Isoflavanone**	Cajanol	5,4′-dihydroxy-7,2′-dimethoxyisoflavanone	Roots, stem/etiolated stems, leaves & seed	Wei et al. (2013a); Liu et al. (2011); Luo et al. (2010); Dahiya et al. (1984); Duker-Eshun et al. (2004); Marley and Hillocks (2002); Ingham (1979, 1976) Dahiya (1987)
	Cajanone		Roots	Dahiya (1991); Preston (1977)
	2′-O-Methylcajanone		Root bark	Bhanumanti et al. (1979)
**Chalcone**	Pinostrobin chalcone	2′,6′-Dihydroxy-4′-methoxychalcone	Leaves	Cooksey et al. (1982); Wei et al. (2013b)

#### Leaves

Pigeonpea leaves are the richest flavonoid containing organ in the plant and are reported to contain six flavones, two isoflavones, two flavonols, two flavanones, an isoflavanone and the single chalcone.

Leaves are the only part of pigeonpea that are recorded as containing flavones which is likely linked to their role in photoprotection against UV irradiation as has been observed in a number of other plant species (Julkunen-Tiitto et al. 2014). The effects of UV application on post-harvest pigeonpea leaves demonstrated that orientin, luteolin, apigenin and apigenin-6,8-di-*C*-α-ι-arabinopyranoside all increased in concentration in response to varying levels of UV exposure post-harvest compared to the control, i.e. no post-harvest UV exposure (Edwards et al. 2008; Wei et al. 2013b).

Orientin and vitexin are among the most abundant flavonoids in pigeonpea leaves with reported concentrations of 18.82 mg/g and 21.03 mg/g respectively (Wu et al. 2009). With the exceptions of the flavanone, pinostrobin and the anthocyanin, chrysanthemin the concentration of other flavonoids from pigeonpea are variable and typically less than 1 mg/g (Table 2).

**Table 2:** **Concentrations (mg/g of plant material) of flavonoids reported from** ***Cajanus cajan***

**Flavonoid**	**Plant organ**	**Concentrations (mg/g)**	**Reference**
**2′-Hydroxygenistein**	Stem	0.037^a^	Ingham (1976)
**4′-O-methylcajanin**	Stem	0.022^a^	Ingham (1976)
**Apigenin**	Leaves	0.130^b^	Zu et al. (2006)
		0.132^a^	Fu et al. (2008)
		0.159^b^	Wei et al. (2013a)
**Biochanin A**	Leaves	0.405^b^	Wei et al. (2013a)
**Cajanin**	Stem	0.074^a^	Ingham (1976)
**Cajanol**	Stem	0.370^a^	Ingham (1976)
	Leaves	0.369^b^	Wei et al. (2013a)
**Chrysanthemin**		2.250^b^	Lai et al. (2012)
**Formonentin**	Leaves	0.318^b^	Wei et al. (2013a)
**Genistein**	Stem	0.105^a^	Ingham (1976)
**Isorhamnetin**	Leaves	0.091^b^	Zu et al. (2006)
**Luteolin**	Leaves	0.263^b^	Zu et al. (2006)
		0.268^a^	Fu et al. (2008)
**Orientin**	Leaves	18.82^b^	Wu et al. (2009)
**Peonidin 3-glucoside**		0.540^b^	Lai et al. (2012)
**Pinostrobin**	Leaves	3.500^b^	Kong et al. (2010)
		5.548^b^	Wei et al. (2013a)
		30.29^b^	Wu et al. (2009)
**Quercetin**	Leaves	0.082^b^	Zu et al. (2006)
**Vitexin**	Leaves	21.03^b^	Wu et al. (2009)

NB. The highest mean yield (extraction concentration, mg/g) is reported here. ^a^concentration reported from fresh material, ^b^concentration reported from dried material.

Biochanin A and formononetin (also known as biochanin B) are the only isoflavones reported from leaves (Wei et al. 2013a). Isoflavonoids are frequently reported as phytoalexins and are important components in plant defence (Dahiya et al. 1984; Marley and Hillocks 2002; Dahiya 1987). Formononetin has been shown to act as a phytoalexin in the etiolated stems of *C. cajan*, and its primary role may be as a precursor to the primary antifungal compound, the isoflavanone cajanol (Ingham 1976). Pinostrobin chalcone is the sole chalcone described from pigeonpea and its occurrence is only reported from the leaves which were challenged with the necrotic fungus *Botrytis cinerea* (Cooksey et al. 1982); it is also hypothesized to play a role in photo-protection (Wei et al. 2013b).

#### Roots, seedling stems and seed

The roots, stem and seed of pigeonpea almost exclusively contain isoflavonoids. The exception is the flavanone, cajaflavanone which has been reported from the roots (Bhanumati et al. 1978; Dahiya 1991). The isoflavones formononentin, genistein, 2′-hydroxygenistein and cajanin, and the isoflavanone cajanol were isolated from etiolated stems of pigeonpea inoculated with the fungus *H. carbonum* while being restricted to trace levels in uninfected plants (Ingham 1976). Cajanol was found to be the primary antifungal compound produced by pigeonpea when tested against different fungal pathogens (Ingham 1976; Marley and Hillocks 2002). The isoflavanone cajanone was also shown to inhibit the growth of the pigeonpea wilt pathogen *Fusarium oxysporum* (Preston 1977).

Investigating the accumulation of phytoalexins showed the presence of cajanol, cajanin, isoprenylated genistein and an unidentified isoflavonoid phytoalexin in pigeon pea after seedlings had been stressed with silver nitrate solution and subjecting them to dark conditions. No phytoalexins were present in stressed seedlings exposed to light (Dahiya 1987).

The isoflavanone cajanone and the flavanone, cajaflavanone have been isolated from root exudates and shown to be responsible for nitrogen nodulation (Dahiya 1991).

Pigeonpea seeds have received little attention. However, seeds that had been soaked, then sliced and incubated under non-sterile conditions accumulated the phytoalexins cajanol, cajanin and two unidentified isoprenylated flavones (Dahiya et al. 1984).

Compounds identified from the surface of pods included three flavonoids, the flavonols quercetin, isoquercitrin (quercetin-3-glucoside) and quercetin 3-methyl ether, and the stilbene, cajaninstilbene acid (CSA). Behavioural studies of larvae of the agricultural pest, *Helicoverpa armigera* demonstrated quercetin 3-methyl ether increased their feeding behaviour while CSA was found to be a feeding deterrent. Pod-boring-resistant cultivars were found to have a higher ratio of CSA to quercetin 3-methyl ether, along with an increase in isoquercitrin and an absence of quercetin on the pod surface, when compared to the commonly cultivated susceptible varieties (Green et al. 2003).

Flavonoid glycosides have received significant attention in health research due to their antioxidant and anticancer properties (March et al. 2006). Pigeonpea contains vitexin and isovitexin, two flavone C-glucosides known to possess antimicrobial effects (Agnese et al. 2001). Orientin, another flavone C-glucoside from pigeonpea, has been shown to possess higher antioxidant activities and more efficient free radical scavenging abilities than vitexin (Wu et al. 2009). Luteolin is a flavone found in high concentration in pigeonpea leaves (Fu et al. 2006) and exhibits a number of pharmacological properties (Lee et al. 2002; Kimata et al. 2000; Perez-Garcia et al. 2000).

There have not been any reports of flavonoid compounds from pigeonpea flowers to date.

## Conclusions

The literature about pigeonpea flavonoids spans almost 40 years. However, there are relatively few published studies and from these we presented a total of 27 flavonoids across seven classes. Despite the importance of pigeonpea as an economic crop for both human and animal nutrition, we show here that the knowledge of flavonoids from pigeonpea and their potential application is largely undeveloped. In particular, there is limited understanding of how the flavonoid profile of pigeonpea affects interactions with insect pest species. For example, cultivated varieties of pigeonpea are susceptible to pod-boring insects, such as *Helicoverpa armigera*, a major agricultural pest, which feed on all plant parts including the seed (Jadhav et al. 2012). However, despite flavonoids being found in the seeds of most plants and coloured flowers, we could only find one published study of flavonoids in pigeonpea seed and none reporting flavonoids from flowers. Based on evidence that suggests specific flavonoids on the surface of the pods affect feeding behaviour (Green et al. 2003) we suggest extending research to include analysis, detection and measurement of the levels of these compounds in all plant parts (particularly flowers). This could be useful to develop varieties of pigeonpea that have increased resistance to pests such as *Helicoverpa* thereby maximising yields.

We suggest that further work exploiting the advances in technology used to isolate, characterise and quantify flavonoids could be applied to enhance plant breeding and allow the agricultural production potential of pigeonpea to be realised.

## References

[CR1] Agnese AM, Perez C, Cabrera JL (2001). *Adesmia aegiceras*: antimicrobial activity and chemical study. Phytomedicine.

[CR2] Ashidi JS, Houghton PJ, Hylands PJ, Efferth T (2010). Ethnobotanical survey and cytotoxicity testing of plants of South-western Nigeria used to treat cancer, with isolation of cytotoxic constituents from *Cajanus cajan* Millsp leaves. J Ethnopharmacol.

[CR3] Baker GH, Tann CR (2013). Mating of *Helicoverpa armigera* (Lepidoptera: Noctuidae) moths and their host plant origins as larvae within Australian cotton farming systems. Bull Entomol Res.

[CR4] Bhanumanti S, Chhabra SC, Gupta SR, Krishnamoorthy V (1979). 2′-O-methylcajanone: a new isoflavanone from *Cajanus cajan*. Phytochemistry.

[CR5] Bhanumati S, Chhabra SC, Gupta SR, Krishnamoorthy V (1978). Cajaflavanone: a new flavanone from *Cajanus cajan*. Phytochemistry.

[CR6] Bhanumati S, Chhabra SC, Gupta SR (1979). Cajaisoflavone, a new prenylated isoflavone from *Cajanus cajan*. Phytochemistry.

[CR7] Bhanumati S, Chhabra SC, Gupta SR, Krishnamoorthy V (1979). New isoflavone glucoside from *Cajanus cajan*. Phytochemistry.

[CR8] Chen ML, Hu W, Zhang C, Fang Y (2011). High performance liquid chromatography for the determination of flavonoids. J Chin Pharmaceut Sci.

[CR9] Cooksey CJ, Dahiya JS, Garratt PJ, Strange RN (1982). Two novel stilbene-2-carboxylic acid phytoalexins from *Cajanus cajan*. Phytochemistry.

[CR10] Dahiya JS (1987). Reversed-phase high-performance liquid chromatography of *Cajanus cajan* phytoalexins. J Chromatogr A.

[CR11] Dahiya JS (1991). Cajaflavanone and cajanone released from *Cajanus cajan* (L. Millsp.) roots induce nod genes of *Bradyrhizobium* sp. Plant Soil.

[CR12] Dahiya JS, Strange RN, Bilyard KG, Cooksey CJ, Garratt PJ (1984). 2 isoprenylated isoflavone phytoalexins from *Cajanus cajan*. Phytochemistry.

[CR13] Duker-Eshun G, Jaroszewski JW, Asomaning WA, Oppong-Boachie F, Christensen SB (2004). Antiplasmodial constituents of *Cajanus cajan*. Phytother Res.

[CR14] Dykes L, Seitz LM, Rooney WL, Rooney LW (2009). Flavonoid composition of red sorghum genotypes. Food Chem.

[CR15] Edwards WR, Hall JA, Rowlan AR, Schneider-Barfield T, Sun TJ, Patil MA, Pierce ML, Fulcher RG, Bell AA, Essenberg M (2008). Light filtering by epidermal flavonoids during the resistant response of cotton to Xanthomonas protects leaf tissue from light-dependent phytoalexin toxicity. Phytochemistry.

[CR16] Emerenciano VP, Militao J, Campos CC, Romoff P, Kaplan MAC, Zambon M, Brant AJC (2001). Flavonoids as chemotaxonomic markers for Asteraceae. Biochem Syst Ecol.

[CR17] Falcone Ferreyra ML, Rius SP, Casati P (2012). Flavonoids: biosynthesis, biological functions, and biotechnological applications. Front Plant Sci.

[CR18] Fu YJ, Zu YG, Liu W, Efferth T, Zhang NJ, Liu XN, Kong Y (2006). Optimization of luteolin separation from pigeonpea *Cajanus cajan* (L.) Millsp. leaves by macroporous resins. J Chromatogr A.

[CR19] Fu YJ, Zu YG, Liu W, Hou CL, Chen LY, Li SM, Shi XG, Tong MH (2007). Preparative separation of vitexin and isovitexin from pigeonpea extracts with macroporous resins. J Chromatogr A.

[CR20] Fu YJ, Liu W, Zu YG, Tong MH, Li SM, Yan MM, Efferth T, Luo H (2008). Enzyme assisted extraction of luteolin and apigenin from pigeonpea *Cajanus cajan* (L.) Millsp. leaves. Food Chem.

[CR21] Green PWC, Stevenson PC, Simmonds MSJ, Sharma HC (2003). Phenolic compounds on the pod-surface of pigeonpea, *Cajanus cajan*, mediate feeding behavior of *Helicoverpa armigera* larvae. J Chem Ecol.

[CR22] Ingham JL (1976). Induced isoflavonoids from fungus-infected stems of pigeon pea (*Cajanus cajan*). ZNaturforsch(C).

[CR23] Ingham JL (1979). A revised structure for the phytoalexin cajanol. ZNaturforsch(C).

[CR24] Jadhav DR, Nalini M, Abhishek R, Dilip P (2012). Effect of some flavonoids on survival and development of *Helicoverpa armigera* (Hubner) and *Spodoptera litura* (Fab) (Lepidoptera: Noctuidae). Asian J Agr Sci.

[CR25] Julkunen-Tiitto R, Nenadis N, Neugart S, Robson M, Agati G, Vepsäläinen J, Zipoli G, Nybakken L, Winkler B, Jansen MAK (2014) Assessing the response of plant flavonoids to UV radiation: an overview of appropriate techniques. Phytochem Rev. doi:10.1007/s11101-014-9362-4

[CR26] Kimata M, Inagaki N, Nagai H (2000). Effects of luteolin and other flavonoids on IgE-mediated allergic reactions. Planta Med.

[CR27] Kong Y, Zu YG, Fu YJ, Liu W, Chang FR, Li J, Chen YH, Zhang S, Gu CB (2010). Optimization of microwave-assisted extraction of cajaninstilbene acid and pinostrobin from pigeonpea leaves followed by RP-HPLC-DAD determination. J Food Compos Anal.

[CR28] Lai YS, Hsu WH, Huang JJ, Wu SC (2012). Antioxidant and anti-inflammatory effects of pigeon pea (*Cajanus cajan* L.) extracts on hydrogen peroxide- and lipopolysaccharide-treated RAW264.7 macrophages. Food Funct.

[CR29] Lee LT, Huang YT, Hwang JJ, Lee PPH, Ke FC, Nair MP, Kanadaswami C, Lee MT (2002). Blockade of the epidermal growth factor receptor tyrosine kinase activity by quercetin and luteolin leads to growth inhibition and apoptosis of pancreatic tumor cells. Anticancer Res.

[CR30] Liu XL, Zhang XJ, Fu YJ, Zu YG, Wu N, Liang L, Efferth T (2011). Cajanol inhibits the growth of *Escherichia col*i and *Staphylococcus aureu*s by acting on membrane and DNA damage. Planta Med.

[CR31] Luo M, Liu X, Zu YG, Fu YJ, Zhang S, Yao LP, Efferth T (2010). Cajanol, a novel anticancer agent from Pigeonpea *Cajanus cajan* (L.) Millsp. roots, induces apoptosis in human breast cancer cells through a ROS-mediated mitochondrial pathway. Chem-Biol Interact.

[CR32] March RE, Lewars EG, Stadey CJ, Miao X-S, Zhao X, Metcalfe CD (2006). A comparison of flavonoid glycosides by electrospray tandem mass spectrometry. Int J Mass Spectrom.

[CR33] Marley PS, Hillocks RJ (2002). Induction of phytoalexins in pigeonpea (*Cajanus cajan*) in response to inoculation with *Fusarium udum* and other treatments. Pest Manag Sci.

[CR34] Nicholson RA, David LS, Le Pan R, Liu XM (2010). Pinostrobin from *Cajanus cajan* (L.) Millsp inhibits sodium channel-activated depolarization of mouse brain synaptoneurosomes. Fitoterapia.

[CR35] Perez-Garcia F, Adzet T, Canigueral S (2000). Activity of artichoke leaf extract on reactive oxygen species in human leukocytes. Free Radic Res.

[CR36] Preston NW (1977). Cajanone: an antifungal isoflavanone from *Cajanus cajan*. Phytochemistry.

[CR37] Satyavathi VV, Prasad V, Khandelwal A, Shaila MS, Sita GL (2003). Expression of hemagglutinin protein of Rinderpest virus in transgenic pigeon pea *Cajanus cajan* (L.) Millsp. plants. Plant Cell Rep.

[CR38] Saxena KB (2010). Quality nutrition through pigeonpea—a review. Health.

[CR39] Singh U, Jain KC, Jambunathan R, Faris DG (1984). Nutritional quality of vegetable pigeon peas *Cajanus cajan* (L.) Millsp: mineral and trace elements. J Food Sci.

[CR40] Tomas-Barberan FA, Martos I, Ferreres F, Radovic BS, Anklam E (2001). HPLC flavonoid profiles as markers for the botanical origin of European unifloral honeys. J Sci Food Agr.

[CR41] Van der Maesen LJG (1985). *Cajanus* DC. and *Atylosia* W. & A. (Leguminosae): A Revision of all Taxa Closely Related to the Pigeon Pea, With Notes on Other Related Genera within the Subtribe Cajaninae, vol 85.

[CR42] Van der Maesen LJG (2003). Cajaninae of Australia (Leguminosae: Papilionoideae). Aust Syst Bot.

[CR43] Wei Z, Zu Y, Fu Y, Wang W, Luo M, Zhao C, Pan Y (2013). Ionic liquids-based microwave-assisted extraction of active components from pigeon pea leaves for quantitative analysis. Sep Purif Technol.

[CR44] Wei ZF, Luo M, Zhao CJ, Li CY, Gu CB, Wang W, Zu YG, Efferth T, Fu YJ (2013). UV-induced changes of active components and antioxidant activity in postharvest pigeon pea [*Cajanus cajan* (L.) Millsp.] leaves. J Agric Food Chem.

[CR45] Wu N, Fu K, Fu YJ, Zu YG, Chang FR, Chen YH, Liu XL, Kong Y, Liu W, Gu CB (2009). Antioxidant activities of extracts and main components of Pigeonpea [*Cajanus cajan* (L.) Millsp.] leaves. Molecules.

[CR46] Zu YG, Fu YJ, Liu W, Hou CL, Kong Y (2006). Simultaneous determination of four flavonoids in pigeonpea *Cajanus cajan* (L.) Millsp. leaves using RP-LC-DAD. Chromatographia.

